# Draft whole-genome sequence of *Brevibacterium casei* strain isolated from a bloodstream infection

**DOI:** 10.1007/s42770-020-00236-x

**Published:** 2020-02-17

**Authors:** Alina Olender, Paweł Rutyna, Marcin Niemcewicz, Agnieszka Bogut, Marzanna Ciesielka, Grzegorz Teresiński

**Affiliations:** 1 Chair and Department of Medical Microbiology, Medical University of Lublin, Lublin, Poland; 2 Biological Threats Identification and Countermeasure Centre, Military Institute of Hygiene and Epidemiology, Puławy, Poland; 3 Chair and Department of Forensic Medicine, Medical University of Lublin, Lublin, Poland

**Keywords:** *Brevibacterium casei*, Infection, Genome, Sequencing

## Abstract

Despite its low virulence potential and a commensal lifestyle as a member of the human skin microbiota, *Brevibacterium casei* has been increasingly reported as an opportunistic pathogen, especially in immunocompromised patients. Here, we present the draft genome sequence of the S51 strain isolated from a bloodstream infection. To the best of the authors’ knowledge, this is the first report of the draft genome sequence of the *B. casei* strain isolated from the clinical infection. The strain was identified using phenotypic and molecular methods and subsequently sequenced using the next-generation sequencing. The draft whole genome was assembled de novo, automatically annotated by Rapid Annotations using Subsystems Technology (RAST) server and scrutinized to predict the presence of virulence, resistance, and stress response proteins. The genome size of the S51 strain was 3,743,532 bp and an average G+C content was 68.3%. The predicted genes included 48 genes involved in resistance to antibiotics (including vancomycin, fluoroquinolones, and beta-lactams) and toxic compounds (heavy metals), 16 genes involved in invasion and intracellular resistance (*Mycobacterium* virulence operons), and 94 genes involved in stress response (osmotic, oxidative stress, cold and heat shock). ResFinder has indicated the presence of a beta-lactamase, and a phenotypic analysis showed resistance to penicillin. This whole-genome NGS project for the S51strain has been deposited at EMBL/GenBank under the accession no. QNGF00000000.

**Electronic supplementary material:**

The online version of this article (10.1007/s42770-020-00236-x) contains supplementary material, which is available to authorized users.

The genus *Brevibacterium* along with other bacterial genera, including *Arcanobacterium*, *Arthrobacter*, *Cellulomonas*, *Cellulosimicrobium*, *Corynebacterium* (non-*diphtheriae*), *Curtobacterium*, *Dermabacter*, *Exiguobacterium*, *Helcobacillus*, *Janibacter*, *Knoellia*, *Leifsonia*, *Microbacterium*, *Pseudoclavibacter*, and *Trueperella*, demonstrate morphologic and biochemical features which fit in the coryneform classification [1]. This term encompasses aerobically growing, asporogenous, non-partially acid-fast, irregularly shaped small gram-positive rods [2]. Within recent years, there have been an increasing number of case reports claiming an association of coryneform bacteria with human diseases [2].

The members of the genus *Brevibacterium* are non-motile, non-fastidious, chemoorganotrophic, obligately aerobic, halotolerant (≥ 6.5% NaCl), and catalase positive. Typical habitats for *Brevibacteria* include raw milk and surface-ripened cheese; they have also been found in animal sources. Moreover, these bacteria are considered a part of the microbiota of the human skin and adjacent structures. Presently, the genus *Brevibacterium* includes 40 identified species [https://www.ncbi.nlm.nih.gov/Taxonomy/Browser/wwwtax.cgi?id=1696], with *B. casei* as the most frequently isolated species from otherwise sterile human sites [3].

Brevibacteria have been mainly involved in the etiology of infections in immunocompromised patients and those suffering from severe underlying diseases such as malignancies or endocarditis. Important risk factors for *Brevibacterium* infections include indwelling foreign materials, prosthetic heart valves, or continuous ambulatory peritoneal dialysis catheters [2–6].

In this study, we report the draft genome of *B. casei* S51 isolated from a bloodstream infection. This is, to the best of the authors’ knowledge, the first report of the draft genome sequence of the *B. casei* strain isolated from the clinical infection.

The bacterial growth was detected in three out of four peripheral venous blood cultures (BacT/Alert system blood culture bottles [bioMérieux, France]) obtained from a 50-year-old male. The primary focus of the infection could not be identified. The empirical treatment with intravenous vancomycin was administered in the patient.

Positive cultures of the bacteremic strain were subsequently subcultured onto the Columbia agar supplemented with 5% sheep blood (bioMérieux, France). The isolates appeared as Gram-positive, club-shaped, slightly curved rods. They produced whitish grey colonies with a distinctive cheese-like odour characteristic of the genus *Brevibacterium*.

Preliminary identification of the species using single colonies of each cultured bacterial isolate was performed using the API Coryne system (bioMérieux, France). The result was confirmed by the PCR amplification of the 16S rRNA gene using bacterial universal primers followed by the DNA sequencing on both strands [7]. The consensus sequence was compared using the Basic Local Alignment Search Tool (BLAST).

Antibiotic susceptibility of the strain was determined using MIC Test Strips (Liofilchem, Italy) on the Mueller-Hinton agar supplemented with 5% defibrinated horse blood and 20 mg/l β-NAD (bioMérieux, France). The tested antimicrobials included penicillin, clindamycin, imipenem, meropenem, ciprofloxacin, vancomycin, teicoplanin, tetracycline, gentamicin, chloramphenicol, and trimethoprim/sulfamethoxazole. The methodology was based on the European Committee on Antimicrobial Susceptibility Testing (EUCAST) guidelines, applying susceptibility results for *Corynebacterium* species.

Libraries for sequencing were prepared from genomic DNA (kit for isolation of DNA from tissue and cells, Macherey-Nagel GmbH&Co KG, Germany). Optimal quality and concentration of extracted DNA were checked with a NanoDrop 1000 Spectrophotometer (Thermo Scientific) and Qubit 2.0 Fluorometer (Life Technologies). For library preparation, the Nextera XT DNA Library Preparation Kit (Illumina) was used according to the manufacturer’s instructions. The samples were sequenced on an Illumina MiSeq 2 × 300-bp paired-end format. The raw sequencing data was demultiplexed and extracted in fastq format.

Total paired-end reads generated from sequencing were quality trimmed and assembled de novo using SPAdes (3.12.0) [8, 9]. Gene prediction, annotation, and RNA search were performed by Rapid Annotations using Subsystems Technology (RAST) [10].

The consensus sequence of 16S gene (758 bp) was compared using BLAST and revealed 95% query cover and 99.59% identities to *Brevibacterium casei* strain DSM 20657 (Accession no. NR_041996.1). The results of the identification were used to predict the strain to whole-genome sequencing.

A total of 2,036,139 paired-end reads (NGS) were quality trimmed and assembled into 60 contigs with a total length of 3,743,532 bp and an average GC content of 68.3%. The *N*_50_ of contig *B. casei* draft whole genome is 134 kb (L50 = 8), with the longest contig being 610,939 kb.

The automatic annotation of the obtained contigs showed the presence of 3411 open reading frames (ORFs), 3358 protein-coding sequences, and 53 RNAs.

The predicted genes included (Fig. 1):

- 48 genes involved in resistance to antibiotics and toxic compounds;
- 16 genes involved in invasion and intracellular resistance;
- 94 genes involved in stress response.

**Fig. 1:**
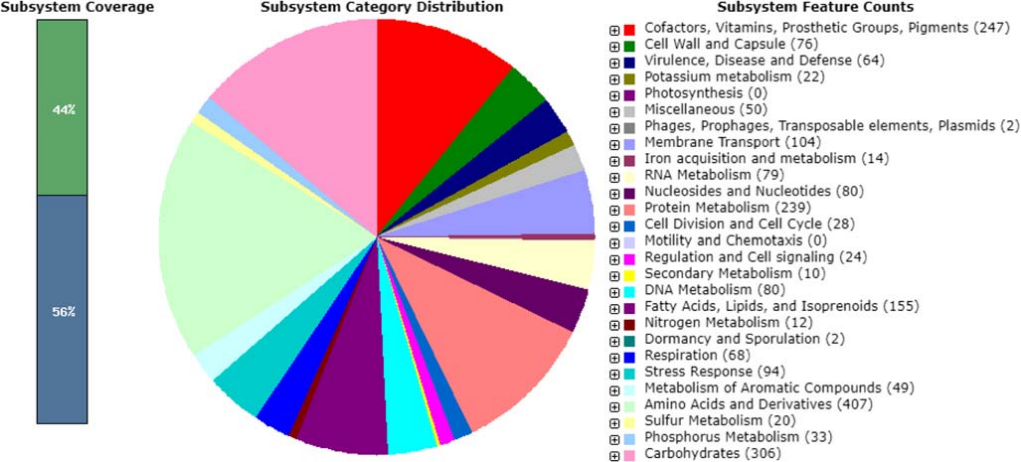
An overview of the subsystem categories of the annotated draft whole-genome *Brevibacterium* casei S51 strain from the RAST server [10]. The pie chart demonstrates the count of each subsystem feature and the subsystem coverage. The bar graph (on the left) determinates the ratio of coding sequences annotated in SEED subsystem features (44%) and outside of the SEED subsystem (56%)

The genome of *B. casei* S51 contains two predicted genes involved in vancomycin resistance (*vanR*, *vanW*), four genes associated with fluoroquinolone resistance (*parC*, *parE*, *gyrA*, *gyrB*), and five genes encoding for beta-lactamases (two genes encoding for beta-lactamase EC 3.5.2.6 and single genes encoding for a beta-lactamase class C and other penicillin-binding proteins, metal-dependent hydrolases of the beta-lactamase superfamily III, and a beta-lactamase class A). The remaining resistance genes included those involved resistance to cobalt-zinc-cadmium, arsenic, chromium compounds, mercury, and copper homeostasis.

Genes involved in invasion and intracellular resistance were represented by five *Mycobacterium* virulence operons including those involved in SSU and LSU ribosomal protein synthesis, DNA transcription, the operon possibly involved in quinolinate biosynthesis, and the operon involved in an unknown function with a Jag protein and YidC and YidD.

Among genes involved in stress responses, 23 were associated with osmotic stress, 46 with oxidative stress, 3 with cold shock (CspA family of proteins), and 14 with heat shock (*dnaK* gene cluster).

Phenotypic analysis of antibiotic susceptibility of the S51 strain revealed sensitivity to all agents tested with the exception of penicillin (MIC 1.5 μg/ml), chloramphenicol (MIC 24 μg/ml), and trimethoprim/sulfamethoxazole (MIC > 32 μg/ml).

Due to the fact that phenotypic analysis of the antibiotic resistance profile of *B. casei* S51 did not correspond to the prediction of resistance genes obtained by RAST, a more detailed investigation of resistance genes using ResFinder 3.2 [11] and CARD (Comprehensive Antibiotic Resistance Database) [12] was performed. Analysis by CARD showed loose hits for all of the predicted resistance genes.

Additional analysis trimmed *.fastq files of S51 strain by ResFinder predicted blaTEM-116 gene with 100% identity and cover responsible for beta-lactam resistance (Accession no. AY425988.1).

According to the available literature data, Brevibacteria are uncommon but important opportunistic pathogens. Starting from 1991, 15 cases of bacteremia caused by this group of bacteria have been reported [3, 5, 6, 13–23]. The predominant species, responsible for 10 out of 15 cases, was *B. casei*. Single reported bacteremia cases were caused by other species including *B. epidermidis* [13], *Brevibacterium massiliense* [23] now designated as *Brevibacterium ravenspurgense* [24], and *Brevibacterium paucivorans* [6] whereas in the two remaining reports, the isolate could not be characterized to the species level. The overwhelming majority of patients reported in the publications cited above suffered from severe underlying conditions including malignancies, AIDS, Crohn’s disease, pulmonary hypertension, diabetes, and chronic heart failure. Indwelling catheters could be identified as crucial risk factors for the development of *Brevibacterium* bacteremia in 12 patients.

It should be noted that phenotypic methods including commonly used biochemical test panels or MALDI-TOF MS technology (matrix assisted laser desorption ionization time of flight mass spectrometry) based on the analysis of the protein composition of microbial cells, may be imperfect for a definite identification of *Brevibacterium* species [14, 23, 24, 26]. Therefore, currently, only molecular methods such as the 16S rRNA gene sequencing enable the reliable identification of Brevibacteria, as reported by Asai et al. [6], Vecten et al. [23], Bernard et al. [24], and Poesen et al. [26].

In spite of an increasing role of *B. casei* as an opportunistic pathogen, its genome has not as yet been sequenced. This is, to the best of the authors’ knowledge, the first report of the draft genome sequence of the *B. case* strain isolated from the clinical bacteremic infection.

The presented genome showed ANI [27] and *is*DDH [28] value of 98.4% and 93.77% to the strain M40 derived from an environmental source (Table 1). The similarity for the reference genome and the *B. casei* S18 strain isolated from the healthy human skin, in turn, was of 98.44% and 93.8% respectively (Table 1). However, in comparison with the reference genome of *B. casei* S18 obtained from the NCBI database, the S51 strain has additional genes potentially important in its pathogenic potential including antibiotic resistance genes and additional stress response genes: cold shock protein (*CspA*) and heat shock protein (*dnaK*).

**Table 1:** Comparison of the genomic feature of *Brevibacterium casei* S51 strain with other *Brevibacterium casei* strains, received from the NCBI database

Strain	DB accession number	Scope	Isolation source	Contigs (scaffolds)	Genome size (bp)	OrthoANI value (%)	*is*DDH value (%)
S51	GCA_000314575.1	Monoisolate	Clinical	60	3,743,532	100	100
S18	GCA_000314575.1	Monoisolate	Human healthy skin	43	3,664,641	98.44	93.80
M40	GCA_001619685.1	Monoisolate	Environmental	88	3,769,110	98.40	93,77
UBA2623	GCA_002339175.1	Multispecies	Environmental	88 (59)	3,687,532	98.37	93,13
UBA 7515	GCA_002476965.1	Metagenomes	Environmental	310 (194)	3,396,412	98.13	93.71
CIP 102111	GCA_900169275.1	Monoisolate	Environmental	24	3,840,753	97.84	91,01
OG2	GCA_002276605.1	Multispecies	Environmental	315	3,885,661	97.78	89.87

The detection of the beta-lactamase *blaTEM-116* encoding gene most probably corresponding to the identified phenotypic resistance against penicillin G has drawn our particular attention. Although *B. casei* has been reported to be uniformly sensitive to tetracycline, gentamicin, glycopeptides, and rifampin [2, 3, 5, 6, 25], decreased susceptibility of the species to beta-lactam agents or clindamycin has been described in previous publications [2, 6, 26].

## Electronic supplementary material

ESM 1 (TXT 788 bytes)

ESM 2 (DOC 25 kb)
