# Supplementary material for: Draft whole-genome sequence of Brevibacterium casei strain isolated from a bloodstream infection
Source: Braz J Microbiol. 2020 Feb 17;51(2):685–9. doi: 10.1007/s42770-020-00236-x (PMC7203332; doi:10.1007/s42770-020-00236-x)
Supplement: Supplementary file 2 — (DOC 25 kb) [file 42770_2020_236_MOESM2_ESM.doc]

Reviewer’s comments:

Minor Comments:  line 33, pg 5 correct spelling of accession

Corrected

Pg 6 line 48 and 53.  It is always difficult to know how to describe taxa which have undergone taxonomic revision.  Ref 21 (alluding to Brevibacterium massiliense) has been found to be a later synonym of B ravenspurgense (ref 22).  Would suggest amending line 53 to something like:  " B. massiliense (21) now designated as B. ravenspurgense (22)…as this would be more accurate

Corrected

Pg. 7 line 30-31…is awkwardly worded, would suggest "its genome has not as yet been sequenced" or alternatively "The genome of B. casei has not as yet been sequenced, in spite of its increasing role as an opportunistic pathogen"

Corrected

*Editor’s comments:*

*Figure 1: Indicate what the colors in the left bar represent (blue and green)*

The bar graph (on the left) determinate the ratio of coding sequences annotated in SEED subsystem features (44%) and outside of the Seed Subsystem (56%)

*The 16S sequence provided as required by the reviewer should be cited where appropriate in the text. I also suggest uploading this supplemental material as a plain text file (.txt) in fasta format.*

The 16S sequence in *.txt file was attached.

Antibiotic resistance analysis: The authors must clarify how they reached the conclusion that 48 genes related to antibiotic resistance are present. This seems to be an exceedingly large number. I don’t understand, for example, the matter with fluoroquinolone resistance. parC, parE, gyrA and gyrB are just conserved genes present in almost every bacterial species. The mere presence of such genes do not indicate antibiotic resistance. Quinolone resistance is a consequence of specific known mutations in some of these genes. It is not clear that this kind of analysis has been performed. If it hasn’t, authors must perform the analyses of the whole genome sequence in a specific resistance database such as ResFinder ([https://linkprotect.cudasvc.com/url?a=https%3a%2f%2fcge.cbs.dtu.dk%2fservices%2fResFinder%2f&c=E,1,p0VyD0RRTlnqNNYeW5X0ise4oec1mpD91RTzoy4zSrTrbIdlJiO3VTSSfd0KJRQQlqCy3vVX784KsJHB0ZUYUzLCdK6OUsTY_B1wVhWq87GRIUU-OFkRwliNYA0r&typo=1](https://linkprotect.cudasvc.com/url?a=https%3A%2F%2Fcge.cbs.dtu.dk%2Fservices%2FResFinder%2F&c=E,1,p0VyD0RRTlnqNNYeW5X0ise4oec1mpD91RTzoy4zSrTrbIdlJiO3VTSSfd0KJRQQlqCy3vVX784KsJHB0ZUYUzLCdK6OUsTY_B1wVhWq87GRIUU-OFkRwliNYA0r&typo=1)) or CARD ([https://linkprotect.cudasvc.com/url?a=https%3a%2f%2fcard.mcmaster.ca&c=E,1,vGVZsPISVmSZh59v9q5mgsURelYb-61cPew_NK8hHnK86sR5wjnQekDaxwdzzGldW2hOxTPA7JczIsUDfFLukTsd6NLrgeTzQxOz29752BNmBg,,&typo=1](https://linkprotect.cudasvc.com/url?a=https%3A%2F%2Fcard.mcmaster.ca&c=E,1,vGVZsPISVmSZh59v9q5mgsURelYb-61cPew_NK8hHnK86sR5wjnQekDaxwdzzGldW2hOxTPA7JczIsUDfFLukTsd6NLrgeTzQxOz29752BNmBg,,&typo=1)). This will result in a more accurate list of antibiotic resistance determinants. If necessary after the analysis, the text and files concerning antibiotic resistance should be updated.

The antibiotic resistance database has been applied; the text and files have been updated.
